# LINKAGE ANALYSIS OF PERCEIVED PHYSICAL FATIGABILITY IN THE LONG LIFE FAMILY STUDY

**DOI:** 10.1093/geroni/igad104.1971

**Published:** 2023-12-21

**Authors:** Emma Gay, Adam Santanasto, Shiow (Rosa) Lin, Mary Wojczynski, Nancy Glynn

**Affiliations:** University of Pittsburgh School of Public Health, Pittsburgh, Pennsylvania, United States; University of Pittsburgh School of Public Health, Pittsburgh, Pennsylvania, United States; Washington University School of Medicine in St. Louis, St. Louis, Missouri, United States; Washington University School of Medicine in St. Louis, St. Louis, Missouri, United States; University of Pittsburgh School of Public Health, Pittsburgh, Pennsylvania, United States

## Abstract

Fatigability, whole-body fatigue anchored to specific tasks, is a highly prevalent characteristic in older adults, but the biological mechanisms underlying perceived physical fatigability are unclear. Genetic studies may identify potential biological pathways contributing to physical fatigability. The family study design of the LLFS provides a unique opportunity to explore variants associated with fatigability using whole-genome linkage analysis. Whole genome sequencing (WGS) was available for N=2462, 496 families (age 73.6±10.5 years, 54.8% women, 99% white. Perceived physical fatigability was measured with the Pittsburgh Fatigability Scale (PFS, 0-50, lower=less severe). Prevalence of lower PFS scores (<15) was 57.9%. Multipoint linkage analysis employing likelihood-based variance components analyses were performed to identify families and genetic loci showing strong evidence for linkage. Based on linkage analysis results, a series of association analyses were performed in the subsets of linked families for PFS Physical scores. Association analyses were conducted using linear mixed models adjusted for age, field center, principal components and familial relatedness. For all families the LOD was 2.13 at chr17:79,549,108. The HLOD of the top 27 families (n=225) was 8.97. There were 8 single nucleotide polymorphisms (SNPs) identified under the linkage peak on chromosome 17. These variants were mapped to 8 different genes involved in regulation of transcription and metabolism, cellular proliferation, signal transduction, protein trafficking, and membrane structure; all important in maintenance of cellular health. Results suggest fatigability in older adults may have multiple molecular etiologies and identified variants may allude to molecular mechanisms to target in future interventions to promote healthy aging.

